# Does Parkinson's Disease Increase the Risk of Atrial Fibrillation? Insights From Electrocardiogram and Risk Scores From a Case-Control Study

**DOI:** 10.3389/fneur.2021.633900

**Published:** 2021-05-12

**Authors:** Mariana Alves, Ana Mafalda Abrantes, Gonçalo Portugal, M. Manuela Cruz, Sofia Reimão, Daniel Caldeira, José M. Ferro, Joaquim J. Ferreira

**Affiliations:** ^1^Serviço de Medicina III, Hospital Pulido Valente, Centro Hospitalar Universitário Lisboa Norte (CHULN), Lisbon, Portugal; ^2^Laboratory of Clinical Pharmacology and Therapeutics, Faculdade de Medicina, Universidade de Lisboa, Lisbon, Portugal; ^3^Faculdade de Medicina, Instituto de Medicina Molecular, Universidade de Lisboa, Lisbon, Portugal; ^4^Medicina 2, Clínica Universitária de Medicina, Hospital Santa Maria, Centro Hospitalar Universitário Lisboa Norte (CHULN), Lisbon, Portugal; ^5^Unidade de Saúde Familiar (USF) Benfica Jardim, Agrupamentos de Centros de Saúde (ACES) Lisboa Norte, Lisbon, Portugal; ^6^Neurological Imaging Department, Centro Hospitalar Universitário Lisboa Norte (CHULN), Lisbon, Portugal; ^7^Faculdade de Medicina, Imaging University Clinic, Universidade de Lisboa, Lisbon, Portugal; ^8^Faculdade de Medicina, Centro Cardiovascular da Universidade de Lisboa, Centro Académico de Medicina de Lisboa (CAML), Universidade de Lisboa, Lisbon, Portugal; ^9^Serviço de Cardiologia, Hospital de Santa Maria, Centro Hospitalar Universitário Lisboa Norte (CHULN), Lisbon, Portugal; ^10^Serviço de Neurologia, Departamento de Neurociências e Saúde Mental, Centro Hospitalar Universitário Lisboa Norte (CHULN), Lisbon, Portugal; ^11^Campus Neurológico Sénior, Torres Vedras, Portugal

**Keywords:** Parkinson's disease, atrial fibrillation, interatrial block, P-wave duration, risk factors, risk scores, case-control study

## Abstract

**Background:** Previous studies suggested that Parkinson's Disease (PD) patients could have an increased risk of atrial fibrillation. However, data supporting this association is not robust. We aimed to compare the potential risk of atrial fibrillation associated with PD in an age and gender matched case-control study, comparing the p-wave indexes from electrocardiograms and clinical risk scores among groups.

**Methods:** A cross-sectional case-control study was performed. All subjects included in the analysis were clinically evaluated and subjected to a 12-lead electrocardiogram. Two blinded independent raters measured the p-wave duration. Subjects were classified as having normal P-wave duration (<120 ms), partial IAB (P-wave duration ≥ 120 ms, positive in inferior leads), and advanced IAB (p-wave duration ≥ 120 ms with biphasic morphology in inferior leads). Atrial fibrillation risk scores (CHARGE-AF, HATCH, and HAVOC) were calculated.

**Results:** From 194 potential participants, three were excluded from the control group due to a previous diagnosis of atrial fibrillation. Comparing the PD patients (*n* = 97) with controls (*n* = 95), there were no statistically significant differences regarding the mean p-wave duration (121 ms vs. 122 ms, *p* = 0.64) and proportion of advanced interatrial block (OR = 1.4, 95%CI = 0.37–5.80, *p* = 0.58). All patients had a low or medium risk of developing atrial fibrillation based on the clinical scores. There were no differences between the PD patients and controls regarding the mean values of CHARGE-AF, HATCH, and HAVOC.

**Conclusions:** Our results do not support the hypothesis that PD patients have an increased risk of atrial fibrillation based on the p-wave predictors and atrial fibrillation clinical scores.

## Introduction

The association of PD with cardiovascular events is still poorly understood (1–3). A systematic review showed a putative increased risk of stroke in PD patients but did not find an increased risk of myocardial infarction or cardiovascular mortality (1). One hypothetic reason for this difference relies on a possible increase of atrial fibrillation risk in PD patients (4, 5). However, the data supporting this association is not robust.

Atrial fibrillation risk can be predicted by the presence of interatrial block (IAB) in the electrocardiogram (ECG), the risk being directly proportional to the p-wave duration, which was probably related to the size of left atrium (6, 7). A systematic review including 16 studies (18,204 patients) reported that advanced IAB, based on the electrocardiogram criteria, significantly predicted new onset AF with HR 2.42 (95% CI = 1.44–4.07).

Due to the clinical complications and economic burden (8) associated with atrial fibrillation, several electrocardiogram criteria such as interatrial block (7) and clinical scoring systems [such as CHARGE-AF (9), HATCH (10), and HAVOC (11)] were developed to aid atrial fibrillation prediction (12).

The authors aimed to compare the potential risk of atrial fibrillation associated with Parkinson's disease in an age and gender matched case-control study, comparing the electrocardiograms and clinical scores among groups.

## Methods

This is an exploratory sub-study included in a cross-sectional case-control study that was performed to evaluate the Carotid Intima Media thickness (CIMT) and other surrogate markers of cardiovascular risk in Parkinson's Disease. All PD patients were diagnosed by a movement disorders specialist according to the UK PDS Brain Bank criteria (13) and had <11 years of disease duration.

Controls were randomly selected, matched to the PD patients according to age and gender. The list of potential controls was split by age and gender in an excel sheet. A number from 0 to 1 was automatically generated and randomly attributed to each line of the excel sheet. The excel content was ordered from highest to lowest, accordingly to the randomized number, and the potential controls were contacted following this sequence. All these subjects were evaluated by the movement disorders specialist to exclude movement disorders.

All participants included in the main study were selected for this sub-study. PD patients were recruited from the Movement Disorders Unit and controls were recruited from the Primary Healthcare Center in the referral area of the hospital.

All examinations were performed with the understanding of and written consent from each subject, with approval from the local ethics committee and in compliance with the national legislation and the Declaration of Helsinki guidelines.

### Assessments

All subjects were evaluated by a physician who performed a detailed clinical evaluation, according to a standardized research protocol.

All participants were invited for a 12-lead electrocardiogram.

Two independent raters, blinded to the diagnosis, previously trained and familiar to this technique, measured the p-wave duration (millimeters) in digitalized ECG images using amplification (Foxit PantomPDF®), as suggested for research purpose (6). The average of the two measurements was used. A trained cardiologist supervised the measurements and resolved any divergences of measurements.

Subjects were divided into three groups: normal P-wave duration (<120 ms), partial IAB (P-wave duration ≥ 120 ms, positive in inferior leads), and advanced IAB (p-wave duration ≥ 120 ms with biphasic morphology in inferior leads) (7). An exploratory cut off of 110 ms for prolonged p-wave was also analyzed (14).

Atrial fibrillation risk scores (CHARGE-AF, HATCH, and HAVOC) were calculated. CHARGE-AF is a 5-year predictive model with C-statistic 0.765; 95% CI = 0.748–0.781, including the variables age, race, height, weight, systolic and diastolic blood pressure, current smoking, use of antihypertensive medication, diabetes, and history of myocardial infarction and heart failure (9). HATCH score includes hypertension, age, stroke or transient ischemic attack, chronic obstructive pulmonary disease, and heart failure. The hazard ratio of each increment of the score is 2.059 (2.027–2.093). The HATCH score range from 0 to 7 and high risk score was defined as ≥5. (10) HAVOC score includes hypertension, age, valvular heart disease, peripheral vascular disease, obesity, congestive heart failure, and coronary artery disease [range 0–214; low risk (scores 0–4), medium risk (5–9), and high risk (10–14)]. The HAVOC score presented an accuracy of 78% and OR = 2–29 (95%CI = 1.37–3.82) for scores above 5 (11).

### Statistics

An exploratory convenience sample including all the patients and controls recruited for a study evaluating surrogate markers of cardiovascular risk in Parkinson's Disease was used in this study. Descriptive statistical methods were used for baseline characteristics. For group comparisons, Chi^2^ test was used to compare nominal variables and Student's *T*-test was used to compare continuous variables. Odds ratio and respective 95% confidence intervals (95%CI) were calculated appropriately.

Data were analyzed using the STATA13.0 (Stata Corporation, College Station, TX, USA). Statistical significance was defined as *p* < 0.05.

## Results

**Figure 1:**
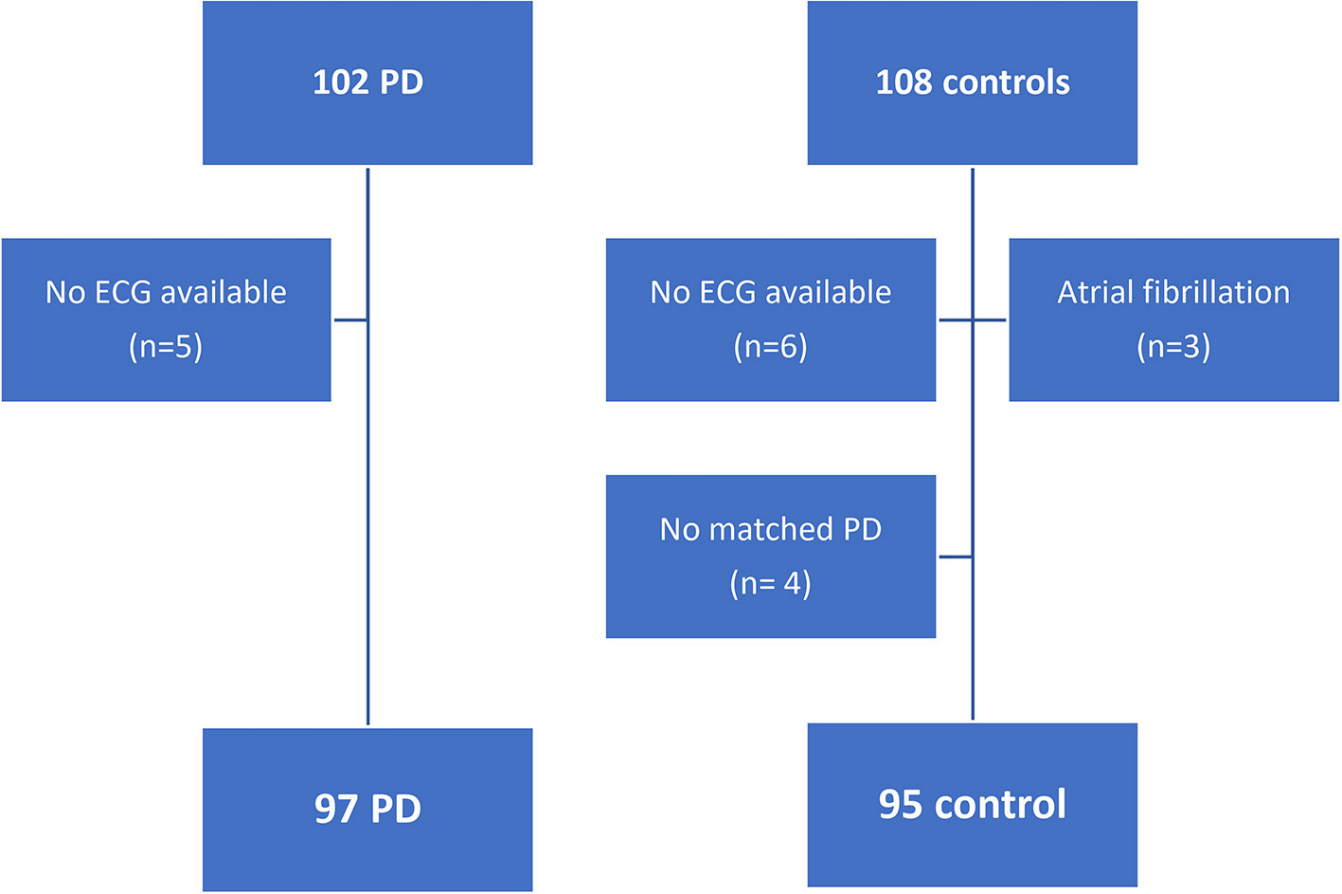
Flowchart of patient's selection.

The study included 210 patients, 102 with PD, and 108 controls (Figure 1). Three participants from the control group were excluded due to the presence of atrial fibrillation. In 11 participants (five PD and six controls), a 12-lead ECG was not available due to a technical failure in transferring the digital electrocardiogram to the medical software or unavailability of the electrocardiograph device at the time the patients were assessed. Risk factors were similar in those patients, except for hypertension which was slightly higher in the PD group (Supplementary Data 1).

PD duration was 4.28 ± 2.72 years, most PD patients presented mild symptoms (mean Hoehn and Yahr 2.03 ± 0.23) and total MDS-UPDRS 43.37 ± 15.98 (range 8–282). Cardiovascular risk factors were similar among groups, except for smoking habits that were lower in PD patients (Table 1).

**Table 1:** Baseline characteristics in Parkinson's disease patients and age- and gender-matched controls.

	**PD (*n* = 97)**	**Control (*n* = 95)**	***P-*value**
Age, years (SD)	65.66 (9.01)	65.59 (9.36)	0.96
Male, *n* (%)	58 (59.8%)	57 (60%)	0.98
Current smoker, *n* (%)	7 (7.2%)	22 (23.4)	0.002
Hyperlipidemia, *n* (%)	38 (39.2)	39 (41.1)	0.79
Diabetes mellitus, *n* (%)	16 (16.5)	12 (12.6)	0.45
Hypertension, *n* (%)	44 (45.4%)	46 (48.4%)	0.67
Coronary heart disease, *n* (%)	5 (5.2%)	4 (4.2%)	0.76
Cerebrovascular disease, *n* (%)	2 (2.1%)	4 (4.2%)	0.39
Peripheral artery disease, *n* (%)	1 (1.0%)	1 (1.1%)	0.99
Heart failure, *n* (%)	2 (2.1%)	0 (0%)	0.16
Chronic obstructive pulmonary disease, *n* (%)	1 (1.0%)	4 (4.2%)	0.17
Heart valve disease, *n* (%)	1 (1.0%)	0 (0%)	0.32
Body mass index (SD)	26.79 (4.30)	27.44 (4.91)	0.33

*SD, standard deviation*.

Regarding the mean p-wave duration and the proportion of interatrial block in our sample, half of the patients presented a prolonged p-wave duration (≥120 ms) and only a minority presented an advanced interatrial block (6%). Comparing the PD patients with controls, there were no statistically significant differences regarding the mean p-wave duration (121 vs. 122 ms, *p* = 0.64) and the proportion of advanced interatrial block (OR = 1.4, 95%CI = 0.37–25.80, *p* = 0.58; Table 2).

**Table 2:** Electrocardiogram outcomes and clinical risk scores predictors of atrial fibrillation.

	**PD (*n* = 97)**	**Control (*n* = 95)**	***P*-value**
**Electrocardiographic outcomes**			
p-wave duration, ms (SD)	120.8 (18.9)	121.9 (17.0)	0.64
**Prolonged p-wave**, ***n*** **(%)**			
≥110 ms	73 (75.3%)	72 (75.8%)	0.93
≥120 ms	47 (48.5%)	49 (51.6%)	0.66
**Interatrial block**, ***n*** **(%)**			
No	50 (51.5%)	46 (48.4%)	0.72
Partial	40 (41.2%)	44 (46.3%)	
Advanced	7 (7.2%)	5 (5.3%)	
**AF clinical risk scores**			
CHARGE-AF (SD)	12.15 (1.12)	12.19 (1.12)	0.82
HATCH (SD)	0.66 (0.73)	0.71 (0.73)	0.67
HAVOC (SD)	1.67 (1.72)	1.62 (1.52)	0.83

*ms, millisecond; SD, standard deviation; AF, atrial fibrillation*.

Considering the clinical risk scores (Table 2), all patients did not have a high score/risk of developing atrial fibrillation. CHARGE-AF score ranged from 8.84 to 14.8. HATCH score ranged from 0 to 3, thus, all the patients were in the low-risk category. HAVOC score ranged from 0 to 7 and only four controls and five PD patients were classified as medium risk. All the others were in the low-risk group.

## Discussion

Our data showed that there was no difference regarding the p-wave indexes and clinical risk scores for developing atrial fibrillation in PD patients compared with age- and gender-matched controls. Many participants presented a prolonged p-wave duration, which is known to be frequent in the older population (7). However, advanced interatrial block, that is more specifically associated with left atrium enlargement and supraventricular arrhythmias (7), was infrequent in both groups. It is also important to emphasize that three controls were excluded from the analysis due to the presence of atrial fibrillation and no PD patient had a previous report of this arrythmia.

Our results are divergent from those published by Canga et al. (4). In that study, the authors compared 51 PD patients with 31 controls and reported an increased proneness for atrial fibrillation development in the PD patients. Besides, they assessed atrial conduction with a different technique, synchronizing echocardiography with a continuous single ECG lead, the control group was not adequately matched. Additionally, PD duration and stage were not reported.

Probably the study that currently supplies more data about PD association with clinical AF was published by Hong et al. (5). The authors used the Taiwanese National Health Insurance Research Database, applying a propensity score model to balance both groups and analyzing transversally (case-control) and longitudinally for 2 years (cohort). In the case-control study, authors concluded that newly diagnosed PD patients were significantly comorbid with AF (OR = 1.15, 95%CI = 1.04–21.28). Elderly (≥65 years old) and women with PD were more likely to have atrial fibrillation. However, in the cohort study, newly diagnosed PD patients had a lower risk of developing AF in the following 2 years (SHR = 0.92, 95% CI = 0.86–20.98). Finally, they suggest that atrial fibrillation could be an early non-motor symptom of PD (5).

Our study is relevant since it focuses on a research gap that is still poorly studied. However, it has limitations. First, in this study, we evaluated indirect electrocardiogram parameters to predict atrial fibrillation. Nevertheless, these patients will be followed to assess the cardiovascular events, in order to also assess the diagnosis of atrial fibrillation. Second, some medications that could interfere with cardiac rhythm and some of the clinical characteristics of risk scores were not actively searched (for example, the chronic obstructive pulmonary disease questionnaires and/or spirometry were not performed in all patients), they were registered as reported in medical records. Third, the HATCH and HAVOC scores were developed mainly to predict atrial fibrillation in post-stroke patients, and their interpretation in this context should be cautious. Additionally, p-wave assessment in a 12-lead electrocardiogram sometimes could be difficult to perform, especially in PD patients with an important resting tremor, which is a transversal limitation/difficulty in all ECG studies in the PD population.

Regarding other methodological limitations, this was an exploratory study which was not powered for this outcome. Additionally, although unaware of which arm corresponds the ECG assessed, examiners were not blinded to the research hypothesis. Lastly, case-control studies are prone to selection bias in respect of the control group, which we tried to overcome using community controls from the referral area of the hospital and randomly inviting the patients by phone contact.

In conclusion, our results do not support the hypothesis that PD patients have an increased risk of atrial fibrillation based on the electrocardiographic predictors and atrial fibrillation clinical scores. Nonetheless, further studies are needed to better understand the link between PD and AF.

## Data Availability Statement

The raw data supporting the conclusions of this article will be made available by the authors, without undue reservation.

## Ethics Statement

The studies involving human participants were reviewed and approved by Ethics committee from Centro Hospitalar Lisboa Norte e Centro Académico Médico de Lisboa. The patients/participants provided their written informed consent to participate in this study.

## Author Contributions

JJF, JMF, SR, and DC conceived and designed the analysis. MC and MA collected the data. AA and GP extracted data. MA and DC performed the analysis. MA wrote first paper draft. All authors discussed the results and contributed to the final manuscript.

## Conflict of Interest

JJF has held consultancy functions with GlaxoSmithKline, Novartis, TEVA, Lundbeck, Solvay, Abbott, Abbvie, BIAL, Merck-Serono, Merz, Ipsen, Biogen, NeuroDerm, Zambon, Sunovion, Affiris, ONO; has received lecture fees from Biogen and BIAL, Sunovion, ONO, Zambon, Abbvie; has received grants from GlaxoSmithKline, Grunenthal, MSD, Allergan, Novartis, Fundação MSD (Portugal), Medtronic and Teva; has been employed by Faculdade de Medicina de Lisboa and CNS - Campus Neurológico Sénior. JMF reports honoraria for speaker, clinical trial coordinator, and advisory board from Boehringer-Ingelheim. DC has participated in educational meetings and/or attended conferences or symposia with Bristol-Myers, Squibb, Bayer, Boheringer Ingelheim, Daiichi Sankyo, Merck Serono, Ferrer, Pfizer, Novartins, and Roche. The remaining authors declare that the research was conducted in the absence of any commercial or financial relationships that could be construed as a potential conflict of interest.

## References

[1] Alves M Caldeira D Ferro JM Ferreira JJ. Does Parkinson's disease increase the risk of cardiovascular events? A systematic review and meta-analysis. Eur J Neurol. (2020) 27:288–96. 10.1111/ene.14076 31454134

[2] Alves M Caldeira D Leal Rato M Duarte GS Ferreira AN Ferro J. Cardiovascular adverse events reported in placebo arm of randomized controlled trials in Parkinson's disease. J Parkinsons Dis. (2020) 10:641–51. 10.3233/JPD-191907 32116264

[3] Alves M Caldeira D Ferro JM Ferreira JJ. Reply to letter: does Parkinson's disease increase the risk of cardiovascular events? A systematic review and meta-analysis. Eur J Neurol. (2019) 27:e12. 10.1111/ene.14125 31710751

[4] Canga Y Emre A Yuksel GA Karataş MB Yelgeç NS Gürkan U. Assessment of atrial conduction times in patients with newly diagnosed Parkinson's disease. Parkinsons Dis. (2018) 2018:2916905. 10.1155/2018/2916905 30123488 PMC6079336

[5] Hong C Chan L Wu D Chen W-T Chien L-N. Association between Parkinson's disease and atrial fibrillation: a population-based study. Front Neurol. (2019) 10:22. 10.3389/fneur.2019.00022 30804869 PMC6370731

[6] Luna AB De Baranchuk A Robledo LAE Roessel AM Martínez-Sellés M. Diagnosis of interatrial block. J Geriatr Cardiol. (2017) 14:161–5. 10.11909/j.issn.1671-5411.2017.03.007 28592957 PMC5460060

[7] Bayés de Luna A Platonov P Cosio FG Cygankiewicz I Pastore C Baranowski R. Interatrial blocks. A separate entity from left atrial enlargement: a consensus report. J Electrocardiol. (2012) 45:445–51. 10.1016/j.jelectrocard.2012.06.029 22920783

[8] Gouveia M Costa J Alarcão J Augusto M Caldeira D Pinheiro L. Burden of disease and cost of illness of atrial fibrillation in Portugal. Port J Cardiol. (2015) 34:1–11. 10.1016/j.repce.2014.08.006 25534665

[9] Alonso A Krijthe BP Aspelund T Stepas KA Pencina MJ Moser CB. Simple risk model predicts incidence of atrial fibrillation in a racially and geographically diverse population: the CHARGE-AF consortium. J Am Heart Assoc. (2013) 2:e000102. 10.1161/JAHA.112.000102 23537808 PMC3647274

[10] Suenari K Chao T-F Liu C-J Kihara Y Chen T-J Chen S-A. Usefulness of HATCH score in the prediction of new-onset atrial fibrillation for Asians. Medicine (Baltimore). (2017) 96:e5597. 10.1097/MD.0000000000005597 28072697 PMC5228657

[11] Ntaios G Perlepe K Lambrou D Sirimarco G Strambo D Eskandari A. External performance of the HAVOC score for the prediction of new incident atrial fibrillation. Stroke. (2020) 51:457–61. 10.1161/STROKEAHA.119.027990 31826729

[12] O'Neal WT Alonso A. The appropriate use of risk scores in the prediction of atrial fibrillation. J Thorac Dis. (2016) 8:E1391–E4. 10.21037/jtd.2016.10.96 27867638 PMC5107446

[13] Hughes AJ Daniel SE Kilford L Lees AJ. Accuracy of clinical diagnosis of idiopathic Parkinson's disease: a clinico-pathological study of 100 cases. J Neurol Neurosurg Psychiatry. (1992) 55:181–4. 10.1136/jnnp.55.3.181 1564476 PMC1014720

[14] Chhabra L Devadoss R Chaubey V Spodick D. Interatrial Block in the Modern Era. Curr Cardiol Rev. (2014) 10:181–9. 10.2174/1573403X10666140514101748 24827803 PMC4040870

