# Supplementary material for: Does Parkinson's Disease Increase the Risk of Atrial Fibrillation? Insights From Electrocardiogram and Risk Scores From a Case-Control Study
Source: Front Neurol. 2021 May 12;12:633900. doi: 10.3389/fneur.2021.633900 (PMC8149901; doi:10.3389/fneur.2021.633900)
Supplement: Supplementary file 1 [file Table_1.DOCX]

**Supplementary data**

**Does Parkinson’s Disease increase the risk of atrial fibrillation? Insights from electrocardiogram and risk scores from a case-control study.**

Mariana Alves, Ana Mafalda Abrantes, Gonçalo Portugal, M Manuela Cruz, Sofia Reimão, Daniel Caldeira, José M Ferro, Joaquim J Ferreira*

**Correspondence:**

Prof. Joaquim J. Ferreira

Laboratório de Farmacologia Clínica e Terapêutica, Faculdade de Medicina de Lisboa, Av. Prof. Egas Moniz, 1649-028 Lisboa, Portugal. Telephone: + 351 21 7802120

jferreira@medicina.ulisboa.pt

Suppl data 1 – Comparing risk factors of the 11 participants without ECG

|  | 192 participants with ECG | 11 participants without ECG | 5 PD | 6 controls | p-value |
| --- | --- | --- | --- | --- | --- |
| **Age** | 65 +/- 10 | 67 +/- 10* | 72+/-11 | 63 +/- 8 | 0.15 |
| **Male** | 115 (60%) | 5 (46%)* | 2 | 3 | 0.74 |
| **Current smoker, n (%)** | 30 (16%) | 2 (18%)* | 0 | 2 | 0.15 |
| **Hyperlipidemia, n (%)** | 76 (40%) | 6 (55%)* | 2 | 4 | 0.38 |
| **Diabetes mellitus, n (%)** | 27 (14%) | 0* | -- | -- | -- |
| **Hypertension, n (%)** | 90 (47%) | 5 (46%)* | 4 | 1 | **0.036** |
| **Coronary heart disease, n (%)** | 9 (5%) | 0* | -- | -- | -- |
| **Cerebrovascular disease, n (%)** | 6 (3%) | 1 (9%)* | 1 | 0 | 0.25 |
| **Peripheral artery disease, n (%)** | 2 (1%) | 2 (18%)****** | 1 | 1 | 0.89 |
| **Heart failure, n (%)** | 2 (1%) | 0* | -- | -- | -- |
| **Chronic Obstructive Pulmonary Disease, n (%)** | 6 (3%) | 0* | -- | -- | -- |
| **Heart valve disease, n (%)** | 1 (0.5%) | 0* | -- | -- | -- |
| **Body Mass Index (SD)** | 27 +/- 5 | 24 +/- 3* | 25 +/- 3 | 24 +/- 3 | 0.41 |

* p>0.05; ** p value < 0.001
